# A Case Report on Enhancing the Dexterity of Finger Prostheses Through the Use of a 3D-Printed Joint Emulator

**DOI:** 10.7759/cureus.69142

**Published:** 2024-09-10

**Authors:** Pragati Agarwal, Seema R Kambala, Surekha R Dubey, Anjali Bhoyar, Ritul Jain, Madhavi S Selukar, Shubham U Tawade

**Affiliations:** 1 Department of Prosthodontics, Sharad Pawar Dental College and Hospital, Datta Meghe Institute of Higher Education and Research, Wardha, IND

**Keywords:** 3d-printed prosthesis, 3d-printed scaffold, finger prosthesis, joint emulator, traumatic amputation

## Abstract

After a traumatic amputation, losing all or part of a finger can be detrimental to one's physical and mental health. Benefits from an esthetically pleasing prosthesis might be psychological, practical, and restorative. Prerequisites for an optimal finger prosthesis include helping the user grasp, absorb, and transfer forces to the hand; the prosthesis should also seem natural and allow for gesture expression. This case study describes how a 3D-printed joint emulator simulates natural joint mobility, significantly enhancing the patient's functional capacity to perform fundamental everyday tasks.

## Introduction

Every year, thousands of people endure terrible hand injuries, which frequently result in the amputation of a finger [1]. Apart from the loss of grip strength and security, severe psychological trauma can result from losing a finger. The most frequent reasons for missing fingers include traumatic injury-related amputations, illnesses, congenital defects, or deformities [2]. Depending on the degree of amputation and the patient's functional requirements, various treatment options are available, including prosthetic fingers, reconstructive surgery, or functionally enhancing adaptive devices. The prosthetic finger seems to be the most practical of them since it can be created to order such that it fits comfortably and looks natural.

A skilled prosthetist must create many plaster molds of the injured limb as part of the extensive, labor and material-intensive traditional prosthesis production procedure. A hastened production process is required since conventional fabrication techniques cannot keep up with the demand for prosthetics. Current developments in additive manufacturing, or 3D printing, have made it feasible to produce batch-production of inexpensive, personalized upper limb 3D prostheses [3]. The creation and practical use of a 3D-printed joint emulator made especially for a finger prosthesis are described in this case study. A finger prosthesis's joint emulator seeks to replicate the normal range of motion and articulation of a finger joint, improving the user's capacity to carry out tasks requiring precise motions and daily activities. Prosthetists and researchers may now create joint emulators that accurately mimic the biomechanical characteristics of human joints by utilizing the flexibility and accuracy of 3D printing.

## Case presentation

A 50-year-old male patient reported to the Department of Prosthodontics and Crown and Bridge with a chief complaint of partially missing middle and ring finger on his right hand for 15 years. He disclosed losing the digits in a road traffic accident. The patient had undergone the procedure for finger prosthesis fabrication five years back, but he was still unable to carry out the most fundamental activities of everyday life. Therefore, he was not content. The patient demanded a movable and esthetically pleasing finger prosthesis.

The patient was well-oriented and free of any medical history during clinical assessment. The partial amputation involved the middle and distal phalanxes of the right middle and ring fingers. Upon careful examination, the flesh surrounding the amputated fingers showed thicker tissue devoid of any indications of infection or inflammation. After extensive consultation and deliberation of the various possibilities, a hybrid method that integrates traditional craftsmanship and digital advancements was considered. Achieving satisfactory results requires the patient to be involved in choosing the fabrication procedure. Before starting the treatment, informed consent was acquired.

Fabrication of the prosthesis

**Figure 1:**
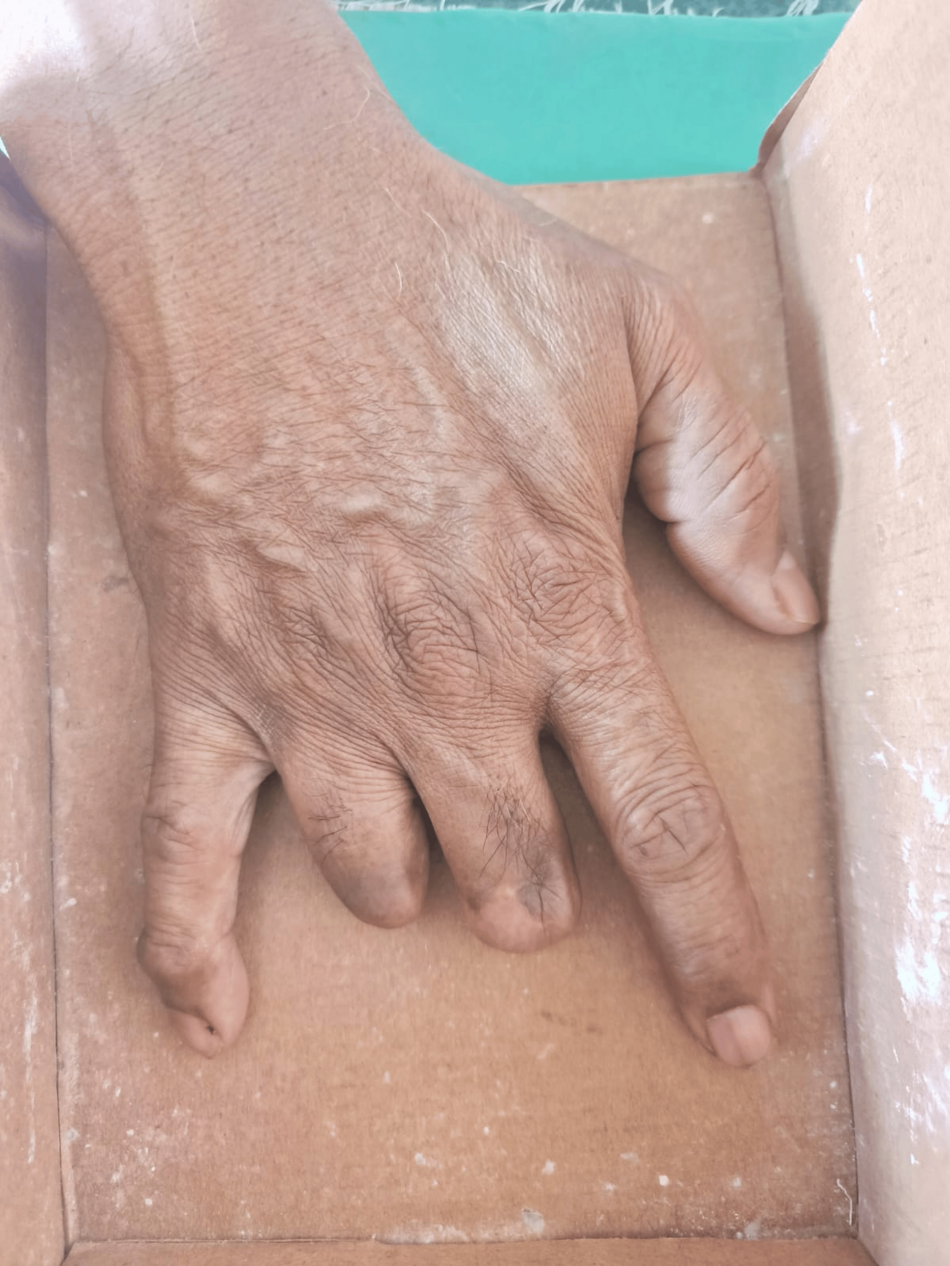
The right hand placed in a cardboard box after applying petroleum jelly

After covering it with petroleum jelly, the right-hand impression was taken using alginate (Zelgan Advanced, Dentsply Sirona, Gurgaon, India) in a cardboard box (Figure 1).

**Figure 2:**
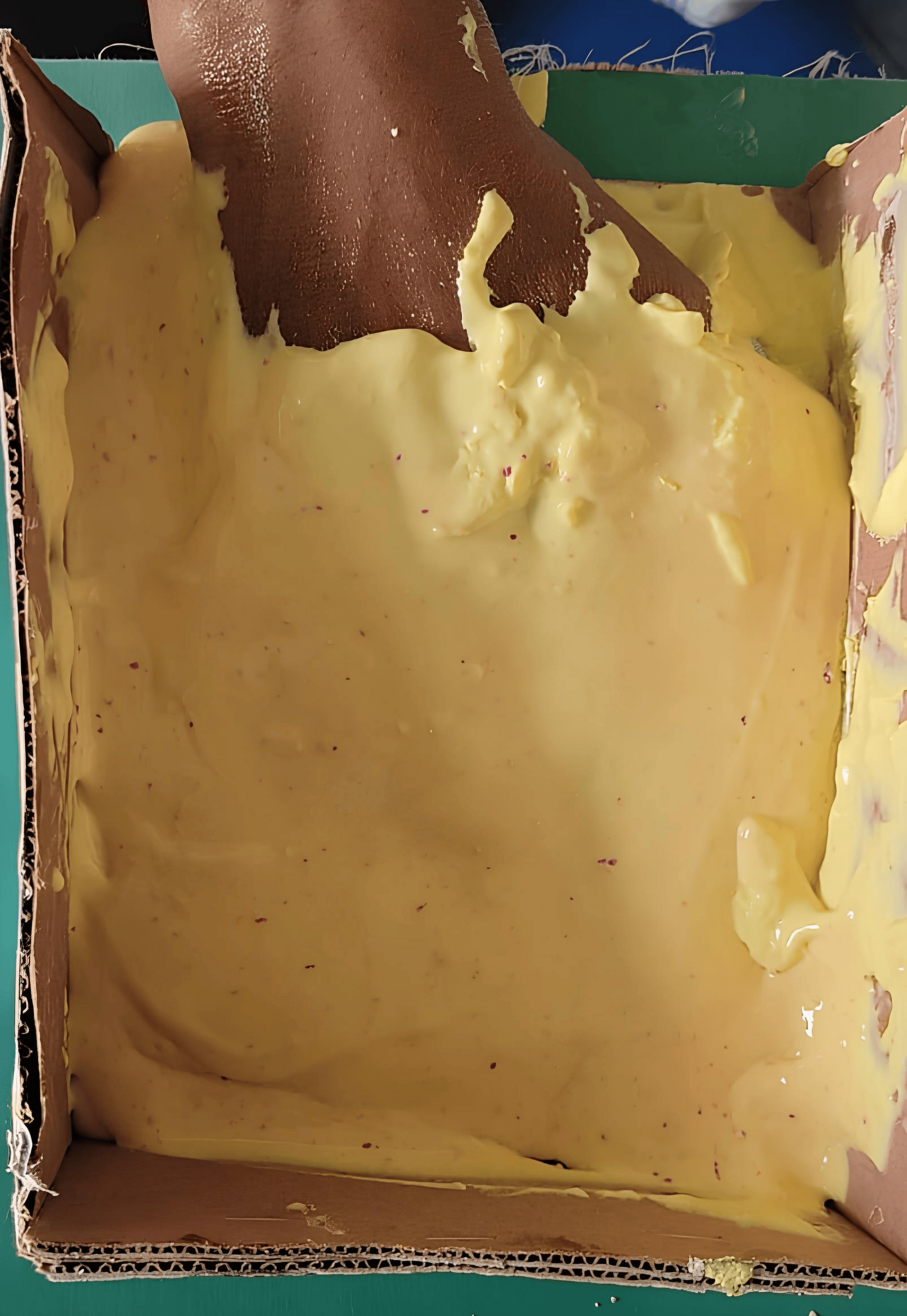
Algniate impression of the right hand

After covering the alginate layer with moist gauze, stapler pins were inserted to ensure retention. To create the body, a layer of dental plaster (Kaldent, Kalabhai Karson, Mumbai, India) was applied on the top of the alginate. Dental stone (Kalrock, Kalabhai Karson) was poured into the alginate impression (Figure 2) to obtain the working cast.

**Figure 3:**
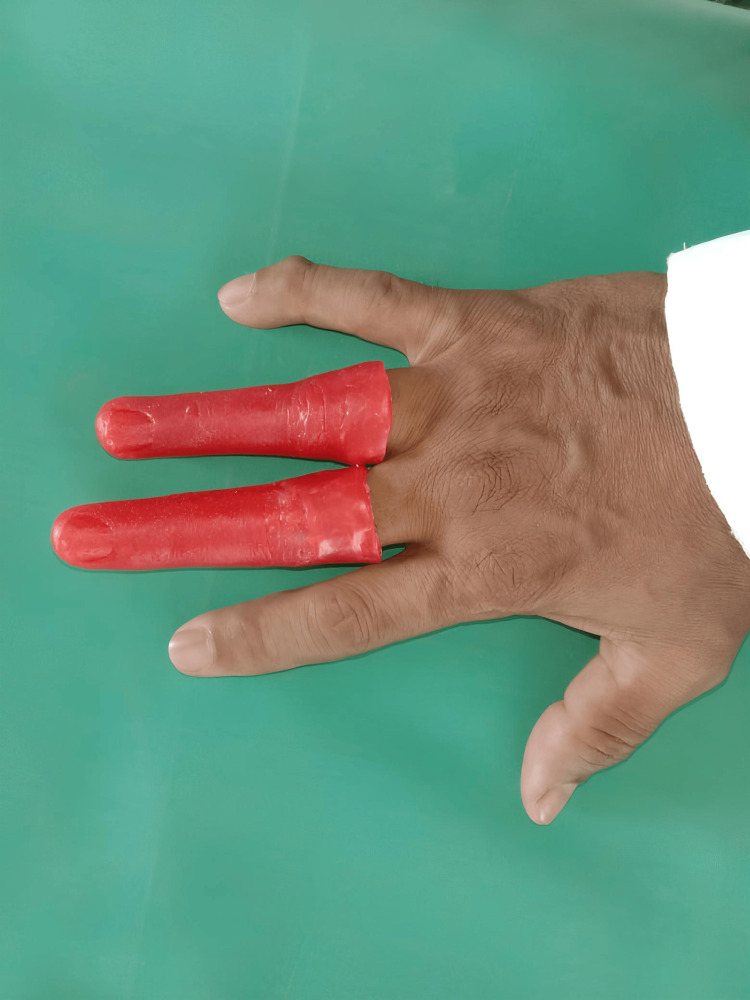
Try-in of the wax pattern

To create the wax pattern, a putty impression of a different individual that resembled the patient's missing finger was created, and it was then poured into wax. The functioning cast finger stump was covered with the wax template, and any required thickness and anatomical adjustments were made. The stability and adaptability of the wax pattern were assessed during the try-in (Figure 3).

**Figure 4:**
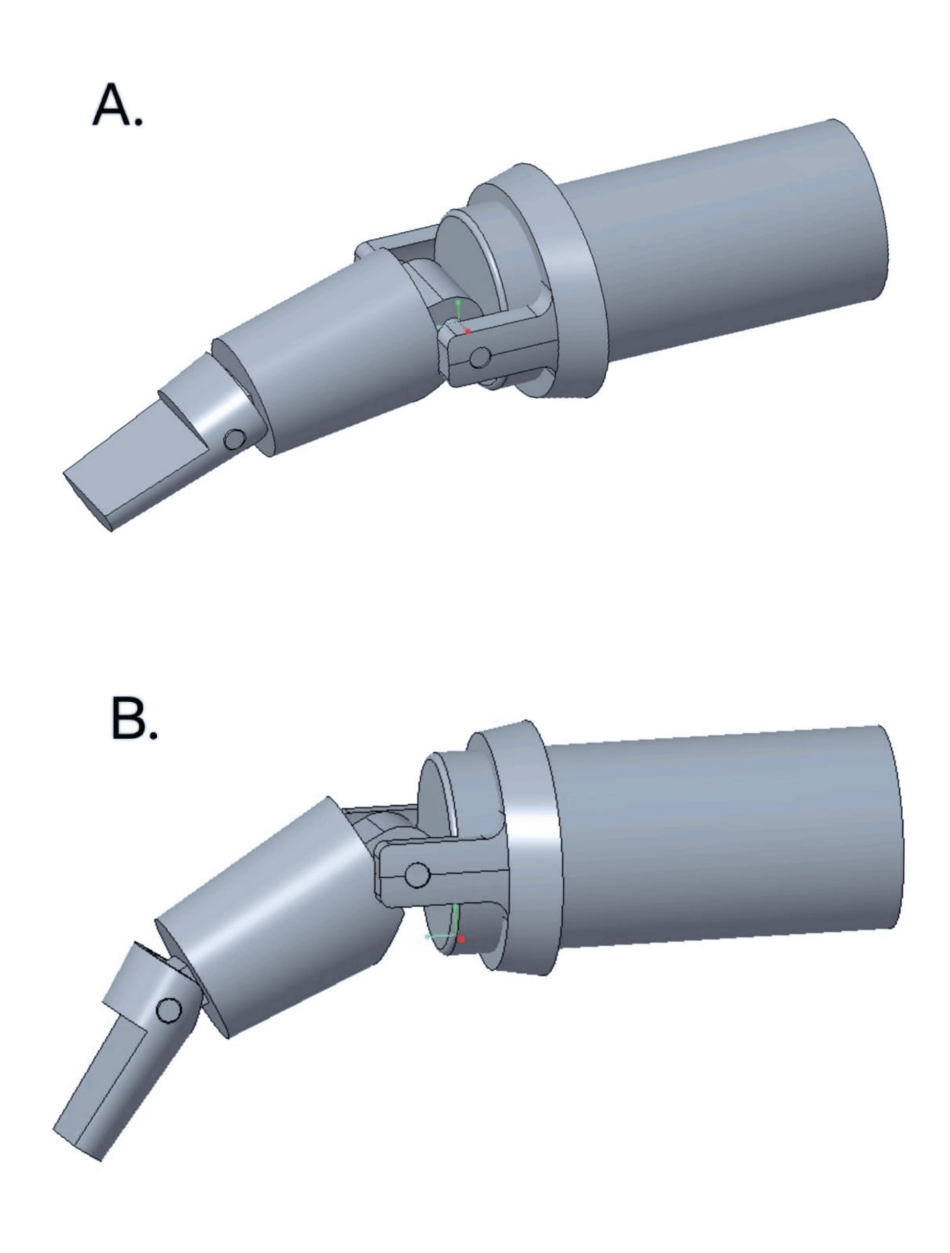
Designing of the joint emulator by CREO 5.0 (PTC Creo, Boston, MA) student version. (A) The design of the joint emulator at 0°. (B) The movement of the joint emulator at 45°

Using a vernier caliper, measurements for the prosthesis were calculated using the wax pattern. The scaffold of the joint emulator was designed as per the measurements in the software (Figure 4).

**Figure 5:**
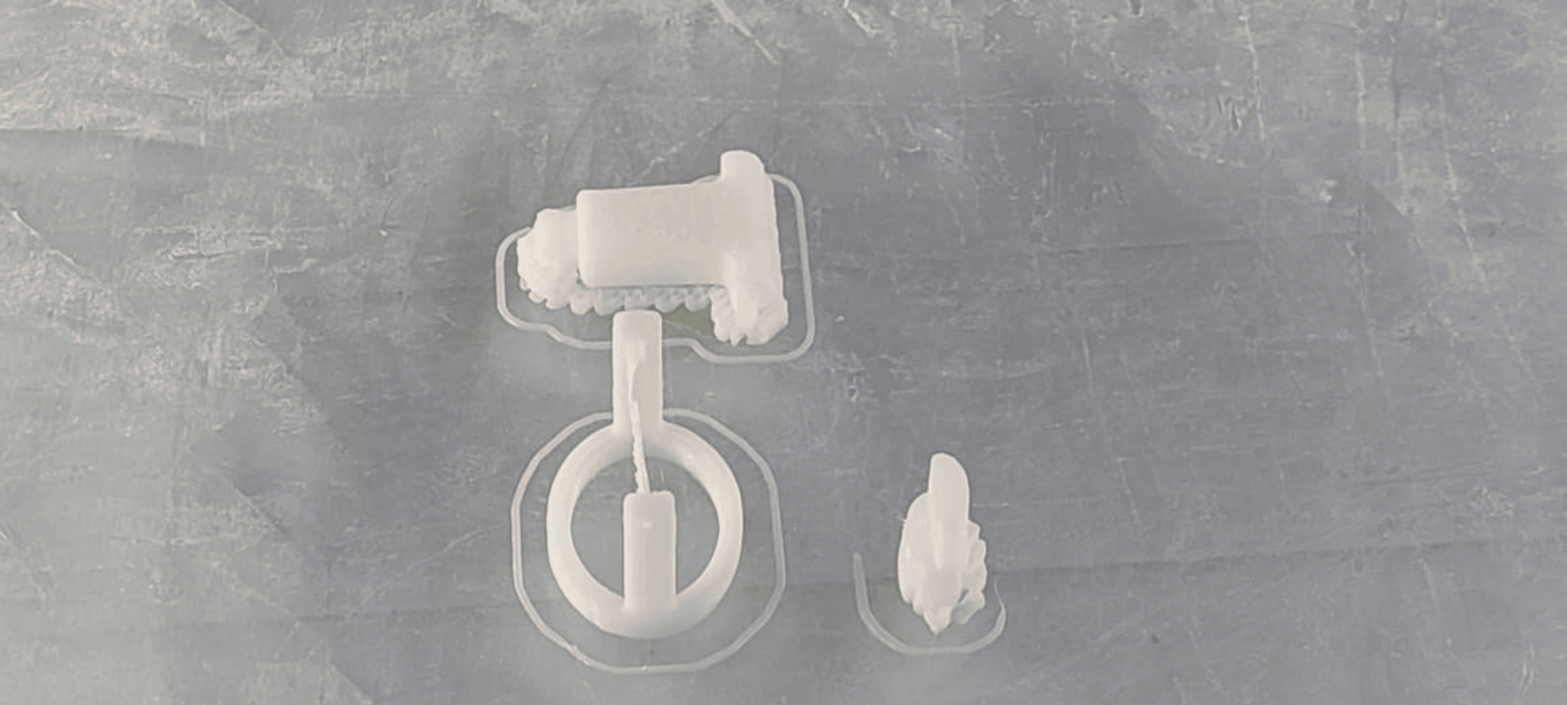
A 3D-printed joint emulator using a fused deposition modeling machine

The scaffold was then 3D-printed using material polylactic acid through the fused deposition modeling technique (Figure 5).

**Figure 6:**
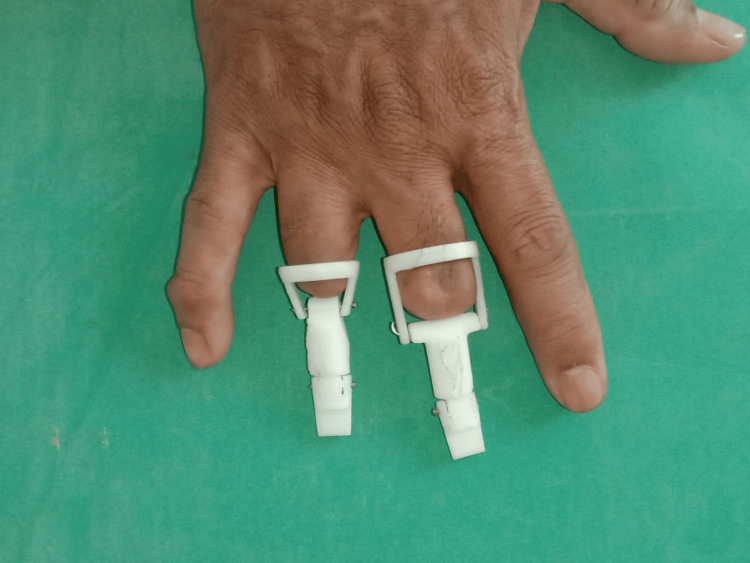
3D-printed joint emulators placed in the patient's fingers

Next, a wax pattern was placed over the 3D-printed scaffold to ensure a proper fit on the finger stump (Figure 6). The flask was filled with the wax design. Throughout the flasking process, two-pour technique was employed. Before the flask was packed, the 3D-printed scaffold was inserted, and the flask was cleaned of wax. Room-temperature vulcanizing silicone was utilized, and a 24-hour cure period was given.

A specially made nail was created and glued into place with cyanoacrylate glue. Color-matched acrylic paints were used for characterization to give components that were not silicone-coated a more esthetically pleasing look. After finishing and polishing, the patient received the finger prosthesis.

Maintenance of prosthesis

The patient received instructions on maintaining their prosthesis when it was delivered. He was given instructions on how to take care of his prosthesis at home, which included using an ultrasoft toothbrush, a mild soap solution, and a warm water irrigant. He was asked back after a month to assess the color stability and functionality of the finger prosthetic. This has also enabled us to evaluate the patient's satisfaction, which was found to be positive, with the prosthesis. The prosthesis demonstrated ideal retention and performance throughout the recalled visit, and the skin surrounding it remained healthy.

## Discussion

Losing any finger significantly influences dexterous people's functionality and appearance. With a suitable prosthesis, most instances involving distal phalanx amputations can return to almost normal functionality [4]. Nevertheless, the particular case under investigation lacked both the middle and distal phalanges, making it more difficult to attain a comparable degree of functionality. According to a Global Burden of Diseases study, India had the highest number of incidences (2.22 million, 95% uncertainty interval, UI, 1.85 to 2.70 million) and prevalences (75.60 million, 95% UI, 69.66 to 81.97 million) of traumatic amputations in 2019 [5]. The data highlights the necessity for creative and efficient rehabilitation programs to help many afflicted people.

In 2020, Mohammadi et al. [6] presented that soft robotic hands with monolithic structures have shown great potential to be used as prostheses due to their advantages of yielding lightweight and compact designs and ease of manufacture.

The literature shows that only two case studies regarding 3D-printed finger prostheses have been reported. A patient-specific body-powered 3D-printed partial finger prosthesis was developed in 2019, and its functional and qualitative aspects were compared with those of a finger prosthesis that was sold commercially. Young et al. thoroughly explained the development process [7]. He concluded that 3D printing has a lot of potential to open up a lot of new medical devices and applications, which could change how future medical devices are made.

In 2023, Vijayan et al. [8] presented the rehabilitation of an amputated index finger employing a novel digital workflow (3D printing). This process eliminated the need for casts and impressions, resulting in a more accurate, efficient, and functionally viable outcome. Motivated by the previously mentioned development, we initiated the process of fabricating an analogous prosthetic through the use of a hybrid methodology that integrates digital and conventional technologies. The goal of this combination is to maximize the prosthesis's fit and functionality by utilizing the advantages of both approaches.

In 2018, Cuellar et al. defined a list of 10 design considerations of nonassembly mechanisms that should be followed to achieve fully functional 3D-printed prosthetic hands [9]. He also revealed in 2020 a bioinspired method for designing and manufacturing articulated fingers for a novel kind of 3D-printed hand prosthesis powered by the user's body and meets fundamental needs [10]. These factors were carefully taken into account when designing the prosthesis that was displayed.

The finger prosthesis's design includes a ring and a joint emulator that replicate the phalanges of the finger's natural joint mechanics. The ring's incorporation improves stability and guarantees correct alignment by offering a snug and comfortable fit around the residual digit. The flexion and extension movements of the phalangeal joints, which range from 0° to 45°, are precisely replicated by the joint emulator, enhancing dexterity and restoring more natural hand functionality. This design lessens the possibility of discomfort or inappropriate loading on the residual stump in addition to making it easier to grasp and manipulate objects.

One drawback, though, was that the middle phalanx's full movement could not be replicated. There is never a flawless prosthesis, and the prosthesis in this case study has esthetic limitations. As the main goal of this prosthesis was to restore functionality, a full silicone oversleeve cannot be made because it was discovered to interfere with the prosthesis's smooth operation. Further improvement in this area will be possible with the use of newer, thinner sheath materials.

## Conclusions

The invention and practical use of the 3D-printed joint emulator for finger prosthesis described in this case study demonstrate important developments in customized healthcare and prosthetic technology. A solution that closely resembles the biomechanical characteristics of natural finger joints was made functional and configurable through the integration of additive manufacturing processes, particularly 3D printing. Additionally, this case study adds to the increasing amount of data demonstrating the viability and effectiveness of 3D-printed prosthetic parts in medical settings. In summary, the incorporation of 3D-printed joint emulators is a potentially significant development in the prosthetics sector, providing tailored and useful solutions that enable people to restore vital hand functions and enhance their overall quality of life.

## References

[1] Kawaiah A, Thakur M, Garg S, Kawasmi SH, Hassan A (2020). Fingertip injuries and amputations: a review of the literature. Cureus.

[2] Pattanaik B, Pattanaik S (2013). Fabrication of a functional finger prosthesis with simple attachment. J Indian Prosthodont Soc.

[3] Zuniga J, Katsavelis D, Peck J, Stollberg J, Petrykowski M, Carson A, Fernandez C (2015). Cyborg beast: a low-cost 3d-printed prosthetic hand for children with upper-limb differences. BMC Res Notes.

[4] Yeo CJ, Sebastin SJ, Chong AK (2010). Fingertip injuries. Singapore Med J.

[5] Yuan B, Hu D, Gu S, Xiao S, Song F (2023). The global burden of traumatic amputation in 204 countries and territories. Front Public Health.

[6] Mohammadi A, Lavranos J, Zhou H (2020). A practical 3D-printed soft robotic prosthetic hand with multi-articulating capabilities. PLoS One.

[7] Young KJ, Pierce JE, Zuniga JM (2019). Assessment of body-powered 3D printed partial finger prostheses: a case study. 3D Print Med.

[8] Vijayan A, Bhatia V, Arora S, Gupta S (2023). Completely digitally fabricated custom functional finger prosthesis. J Indian Prosthodont Soc.

[9] Cuellar JS, Smit G, Zadpoor AA, Breedveld P (2018). Ten guidelines for the design of non-assembly mechanisms: the case of 3D-printed prosthetic hands. Proc Inst Mech Eng H.

[10] Cuellar JS, Plettenburg D, Zadpoor AA, Breedveld P, Smit G (2021). Design of a 3D-printed hand prosthesis featuring articulated bio-inspired fingers. Proc Inst Mech Eng H.

